# Joining of Individual Silver Nanowires via Electrical Current

**DOI:** 10.1007/s40820-014-0001-9

**Published:** 2014-09-13

**Authors:** Arash Vafaei, Anming Hu, Irene A. Goldthorpe

**Affiliations:** 1 Department of Electrical and Computer Engineering, University of Waterloo, 200 University Avenue W, Waterloo, ON N2L 3G1 Canada; 2 Department of Mechanical and Mechatronics Engineering, University of Waterloo, 200 University Avenue W, Waterloo, ON N2L 3G1 Canada; 3 Department of Mechanical, Aerospace and Biomedical Engineering, University of Tennessee Knoxville, 1512 Middle Drive, Knoxville, TN 37996-2210 USA

**Keywords:** Nanowires, Nanowelding, Joule heating

## Abstract

A procedure for joining polyol-synthesized silver nanowires in air using current-induced Joule heat welding is reported. Using a common probe station and photolithographically patterned gold electrodes, the welding process is completed using a common semiconductor analyzer. A unique two-step procedure eliminates the dielectric barrier at the point of contact without damaging the nanowires away from the junction. This procedure is designed for metal–metal contacts where a strong dielectric intermediate layer might exist, which can occur with metals prone to oxidation or corrosion in air, or as a result of the electrode deposition process. Ohmic connections are also established in cases where there is an initial gap between two nanowires.

## Introduction

Nanowires (NWs) are elongated structures with two nanosized dimensions and a third dimension which is typically several hundreds of nanometers or more. Their high aspect ratio and surface area lead to unique properties, which are exploited in applications as diverse as solar cells [1], integrated circuits [2], and bio-sensing [3]. Silver nanowires in particular are of interest since silver has the highest electrical and thermal conductivity of any metal. Silver nanowires have been used in devices such as plasmon carriers [4], sensors [5], and nano-electromechanical systems (NEMS) packaging interconnections [6].

Welding can be used to construct complex structures from the bottom-up out of simple constituents. Connecting nanowires with electrodes and the formation of nanowire interconnects also require ohmic welding. Methods used to weld nanowires include electron beam bombardment [7], ion beam bombardment [8], plasmonic welding [9], soldering [10], cold fusion [11], pulsed laser [12], and Joule heating [13–15]. Of these, Joule heat welding has several advantages. Electrical contact to small structures is already well understood, and the equipment required for this process is readily available and inexpensive. This is in contrast to the other methods which require equipment such as ion or electron guns, lasers, advanced microscopy, and vacuum technology which make them costly and complex. Using Joule heating, one can construct a self-executing process whereby the welding mechanism autonomously stops when the weld is complete. This trait is only shared with certain types of plasmonic welding, where the localized plasmonic excitations decrease once welding is completed [9, 16]. However, because the wavelength required for plasmonic welding is material dependent, a sophisticated light source is required, and there can be difficulty in welding dissimilar materials. Joule heat welding is more versatile and only requires the materials to be capable of conducting current. Joule heating can also be applied regardless of contact direction and is concentrated at the junction, whereas bombardment techniques can cause damage to the nanowire away from the contact point and introduce impurities. For these reasons, Joule heating is chosen here as the method of welding.

For Joule heat welding, electrical contacts to the nanowires must be created. Procedures such as e-beam lithography [17], focused ion beam chemical vapor deposition [18], and micro-actuators [14] have previously been used to contact nanowires. However, these techniques require costly equipment, can induce damage in the nanowires, are slow, and can be difficult to execute. For the purposes of this project then, common photolithography was used to contact the nanostructures. Photolithography is cost effective and widely available, and thousands of electrodes can be deposited in a single run. The combination of photolithography to deposit electrodes, followed by Joule heating to weld individual nanowires together, has not yet been implemented to the best of our knowledge.

The nanowires used in this project were synthesized in solution using a variation of the polyol method [19]. This method allows one to synthesize crystalline silver nanowires cheaply and quickly. Because of the adaptability of this process to large-scale industrial synthesis, this type of nanowire is already being used in commercial devices such as transparent electrodes. Welding of these polyol-synthesized silver nanowires on an individual scale is of great interest and has not previously been reported.

In this paper, photolithography is used to initiate contact to silver nanowire junctions using gold electrodes. Using a common probe station, current is driven through the electrodes and, hence, the nanowires for the purpose of welding the two nanowires together. A welding procedure is prepared and tested for reliable welding of silver nanowires, which destroys intermediate layers and welds the two nanowires. This procedure requires two steps, which is contrary to Joule heating recipes presented previously. Due to a lack of significant intermediate layers, other researchers were able to weld nano-objects together using constant current [13, 14] or constant voltage [15]. This method of welding is insufficient with this type of nanowire due to large initial resistances and breakdown behavior. This process is also applicable to other corrosion-prone nanowires such as copper and aluminum nanowires, as well as for other metal nanowires where fabrication processes introduce intermediate layers at the contacts.

## Experimental

Ag NWs were prepared in a polyol solution using a method modified from the literature [20]. Briefly, 330 mg of polyvinylpyrrolidone or PVP ((C_6_H_9_NO)_n_, K25, M.W. = 24000, Alfa Aesar) and 12.5 mg of silver chloride (AgCl, Alfa Aesar) were mixed with 40 mL of ethylene glycol (EG, Fisher Chemical) inside a round-bottom flask. The mixture was heated to 160–170 °C for 30 min. Then, 110 mg of silver nitrate in 10 mL of ethylene glycol was heated to 160–170 °C and subsequently added to the mixed solution while stirring vigorously for 4 h. The Ag NWs were cleaned using deionized water and centrifuged at 4,000 rpm/s using a 50 mL centrifugation pipe. The clean supernatants were removed from the centrifugation pipes using a pipette, and the excess PVP and ethylene glycol in the Ag NW solution were removed by repeatedly washing using centrifugation. Centrifugation was subsequently used to isolate the nanowires from the byproduct of fabrication. The nanowires had an average diameter of 90 nm. As reported in previous publications [11, 19], and as is common to all polyol-synthesized silver nanowires, the nanowires are single crystalline with five twin planes running along the longitudinal axis. The nanowires are oriented along the {110} crystallographic direction and have a pentagonal cross-section with 5 {100} surface facets.

The nanowires were randomly dispersed onto a SiO_2_ substrate by drop-casting. The wires on the substrate were then further washed with acetone to remove residual PVP. Observations show that compared to annealing, an acetone wash not only removes the PVP more thoroughly, but also avoids structural damage to the nanowires from excess heating [21]. 100 × 100 nm gold electrodes, distanced 6 μm apart, were deposited on top of the nanowires using a standard lift-off photolithography procedure. The electrodes were deposited on top of the nanowires, rather than deposited prior to nanowire deposition, to reduce intermediate layer formation from adsorbates appearing on the gold films [22] and to eliminate wire deformation in the wells between the electrodes [23]. Suitable nanowire junctions with electrodes at the ends were located using optical microscopy and later probed with an Agilent 4155C Semiconductor Analyzer as the current forcing device. The welding was done in air, at room temperature, under normal room lighting conditions.

## Results and Discussion

**Fig. 1:**
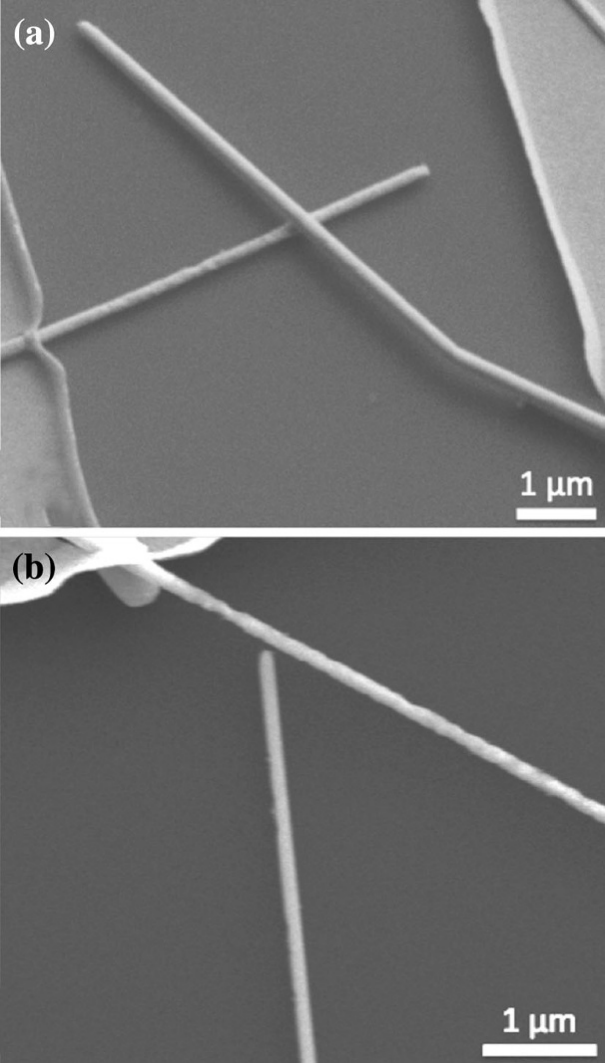
**a** An overlapping junction. The two nanowires provide a path for current to flow between the electrodes. **b** A gap junction. To initiate current flow, silver atoms will have to migrate to close the gap

The configuration of interest for the purposes of this study is two nanowires bridging the gap between two electrodes in series, creating a nanowire–nanowire junction in the middle. Two types of junctions are identified. The first is the overlapped junction where two wires are in contact, or at least appear that way when viewed from above, and are also directly connected to the electrodes. Figure 1a shows an example of this type of junction. The second type of junction is the gap junction (Fig. 1b), where there is a small gap present between one of the points of contact, either at the nanowire–nanowire junction or a nanowire-electrode junction. Since welding nanowires that already have a point of contact, i.e., overlapped, will be more common, the focus of this paper will be on the welding of this type of junction, and gap junctions will be addressed briefly afterward.

### Overlapped Junctions

**Fig. 2:**
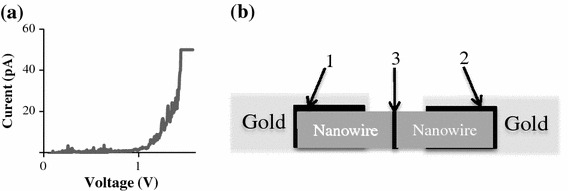
**a** The first voltage ramp forced on an overlapped nanowire junction, showing a surge in current at about 1.5 V. A current limit was set at 50 pA. **b** Each of the three junctions in this device (two between a gold electrode and a silver nanowire, and a third between the two nanowires themselves) contribute to the breakdown. Current will flow when the breakdown voltage of all junctions is breached

The initial resistance of all overlapped nanowire junctions tested was between 1 × 10^12^ and 1×10^14 ^Ω, which at this scale represents an open circuit. Figure 2a shows a typical response when a voltage ramp is performed on such a junction. These devices behave as very high resistance junctions with gradually decreasing resistances until at a certain “surge voltage,” where the current response spikes to very high levels. Almost no samples (less than 1 %) displayed an ohmic response in the beginning. And for cases where the contact was initially ohmic, the contact resistance was very high.

The response shown in Fig. 2a is characteristic of a dielectric sandwiched between two metals, breaking down once a certain voltage is reached. The presence of a layer of dielectric between the nanowire–nanowire contact and the nanowire-electrode contacts would cause the formation of such an energy barrier. These dielectric layers should be taken into account in any current regiment designed to weld the nanowires. A schematic of the system involved is shown in Fig. 2b.

There are several candidates for the identity of the intermediate layers between the contacts in the device. There could be residual polymer (PVP) or photoresist remaining from the synthesis and fabrication process. Or the silver nanowires could exhibit the existence of an oxide or more likely a sulfide layer, as silver nanowires corrode in air [24]. The latter is more likely since it was observed that the resistance of the junction increased over time.

These intermediate layers have to be dealt with in order to achieve welding between two nanowires. One can induce high temperatures in resistive contacts through the electron scattering that occurs with current flow. Since the resistance of the system is highest at the contact points, heating is concentrated there, breaking down the intermediate layers with sufficient temperatures. This heating also locally melts the nanowires, causing a diffusion of ions which leads to welding at the point of contact.

The power dissipated in a resistor due to a constant current or voltage is *I*^*2*^*R* and $\frac{V^{2}}{R}$, respectively. With a sufficiently high constant current, the resistance reduces once a good contact has been formed, and thus the power and the heat are autonomously reduced. Using a constant current is the tactic used in other nanowire Joule heat welding experiments, where thanks to the use of vacuum or a different set of nanowires, no significant intermediate layer hinders the welding process [13–15, 25]. However, due to the very high initial resistance of the junctions in our work, even the lowest setting of a practical current source generates too much power such that nanowires get damaged before welding is complete.

Since power is inversely proportional to resistance when a voltage source is utilized, voltage ramps constitute the ideal source of electrical stimulation at the outset since the high resistances keep the power at low levels. However, while using a voltage source, the contact resistance reduces the power and hence the heat dissipated increases. This means that although voltage can be used to bring the resistance down initially, there will come a point when the resistance is low enough such that small voltage step increases can cause nanowire damage, particularly when the breakdown voltage requirements are reached which is the point where a sharp decrease in resistance occurs.

Because the use of only a current or voltage source causes damage when welding metallic constituents have highly resistive intermediate layers, a unique two-step procedure is designed. A voltage source is initially used to lower the contact resistances, allowing a current source to be subsequently used to complete the weld in a self-executing manner.

**Fig. 3:**
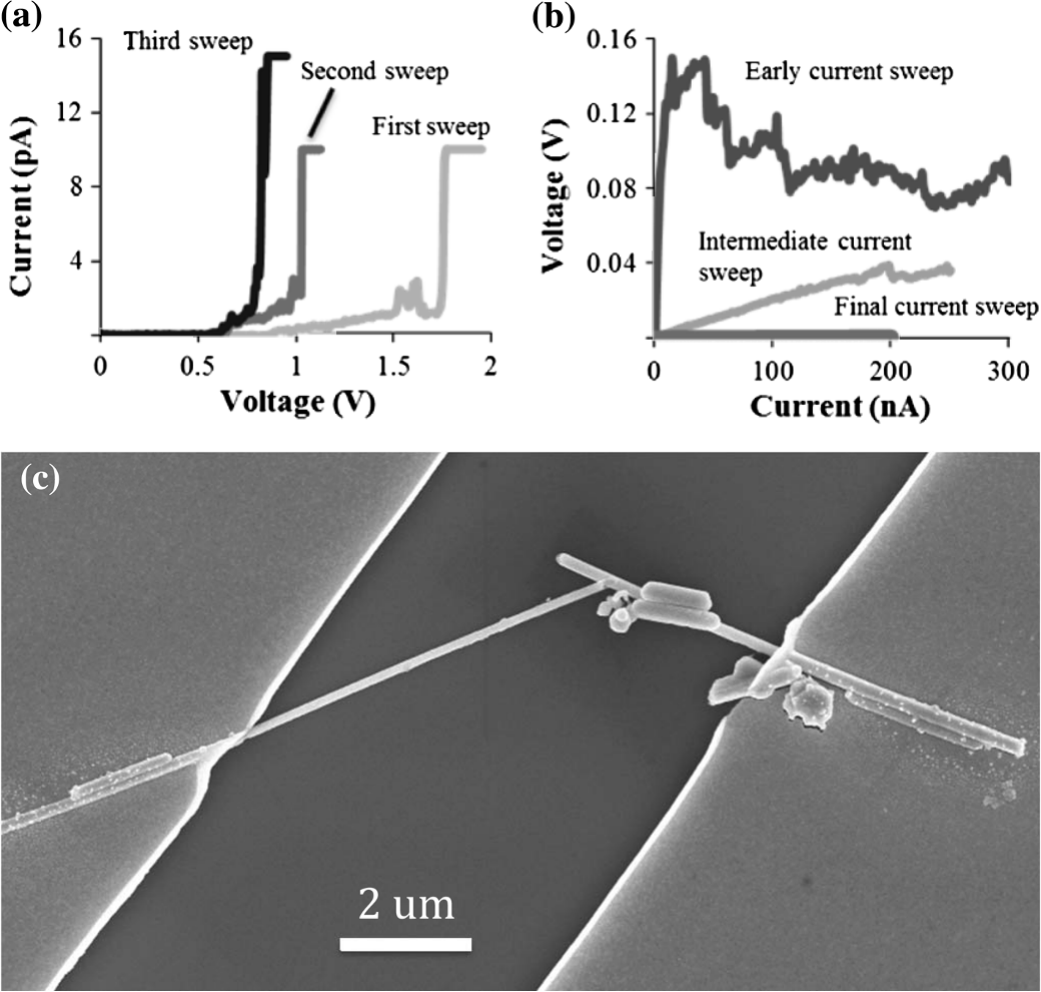
The recorded response of overlapping nanowires to voltage and current sweeps. **a** Voltage sweeps are first used to reduce resistance at the contacts. **b** Current ramp sweeps are used to further decrease resistance and establish an ohmic connection. **c** SEM image after the welding process was completed

By first using a voltage ramp, the potential difference at the junction is increased until the dielectric barriers are breached, leading to an expected sharp increase in current. Using a ramp function makes finding this “surge voltage” quick and practical, without having to incrementally adjust a constant voltage, and adjusting it again should the “surge voltage” change. To avoid damage to the nanowire at this point, a current limit is employed. Each time the “surge voltage” is reached, the spike in power and heat breaks down a part of the intermediate layer leading to a reduction in the contact resistance. When the voltage ramp is repeated, the slope of the *I*–*V* curve increases due to the lower contact resistance, and the current surge is achieved at lower voltages. The ramps are repeated until the resistance is low enough such that forcing a current would not immediately damage the nanowire. The current response of an overlapping nanowire junction to voltage sweeps is shown in Fig. 3a. The point at which a switch can be made to ramping currents is found through experimentation, and is dependent on the type of nanowire and contact fabrication methods used. In this case, once the current began to surge at voltages below 1 V, it was safe to subject the nanowires to currents under 1 nA without significant damage. The purpose of the voltage sweeps is to condition the contacts to prepare them for a current source, since with current a self-executing weld is possible.

As mentioned previously, with driving currents, the power reduces as the resistance decreases. This means that a process can be designed whereby as the welding completes, the heat automatically subsides, leading to a self-executing welding process. As Tohmyoh showed [14], there is a threshold of power at which the heat applied is enough for welding yet low enough not to cause to damage to the nanowires. Current ramps serve as a method of finding the appropriate current quickly, without having to incrementally adjust a constant current measurement. While resistance is still high, small current steps must be used because large steps can burn the nanowire. However, when using small steps, it takes an impractically long time to reach a sufficiently high current to complete the weld (and current-source units often have a maximum limit to the number of measurement steps). That is why multiple current ramps, each with larger step sizes, are thus used to lower the contact resistance step by step. During each current ramp, before welding begins and resistance changes, power and heat increase along with the increasing current. As the heat approaches the breakdown temperature of the intermediate layer, the layer partly dissolves and the resistance decreases, which is observed as a drop in the voltage response. Since the power decreases as the intermediate layer becomes ineffective, the complete dissolution of the intermediate layer does not typically occur until a current on the order of 1 mA is reached. At this point, the heat at the junction is also high enough for local melting of the silver and thus for welding to occur. As found in other works on Joule heat welding of metallic nanowires [14], the power required for welding was not dependent on nanowire length since the resistance of the junction is much larger than the resistance of the nanowires themselves [26]. Once melting is achieved, the junction exhibits a linear voltage–current response. The completion of welding is evident with further measurements. This is done by first leaving the junction to settle without current for several minutes and then running a current ramp. A linear response would confirm the completion of the weld, while a surge response with a voltage spike at the start of the measurement would show an uncompleted weld. The typical electrical response to such current ramp sweeps is shown in Fig. 3b, and an image of a typical overlapped junction after welding is shown in Fig. 3c. The image shows that the morphology of the nanowires is minimally affected away from the junction, including next to either electrodes.

**Fig. 4:**
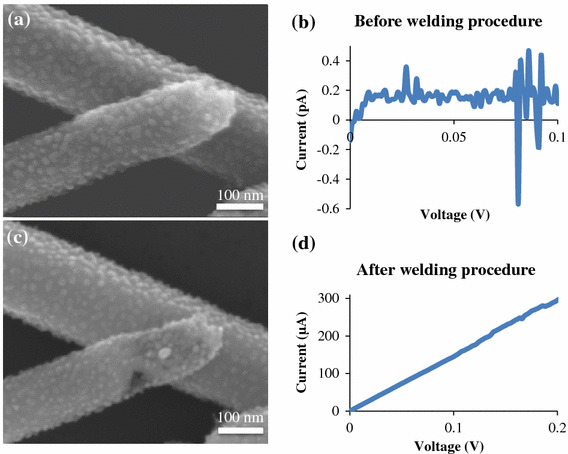
High-magnification images of the junction presented in 3(c), before (**a**), and after (**c**) the welding procedure was applied. **b** and **d** show the corresponding *I*–*V* curves, with the resistance decreasing from 2.4 × 1,012 Ω to 666 Ω

Although welding of silver nanowires synthesized by the polyol method has not been previously reported, it has been shown elsewhere that the temperature of these type of nanowires rises due to Joule heating when current is passed through them [27]. It has also been shown that the resistance of overlapping silver nanowire junctions is much higher than along the nanowires themselves [26], and therefore the heat must be concentrated at the junctions. In the SEM images shown in Figs. 3c and 4, we see that in the post-weld images (Figs. 3c, 4c) the only significant morphology change is at the junction, consistent with the heat being concentrated there. Furthermore, it can be seen in the high-magnification images shown in Fig. 4 that the morphology of the larger nanowire underneath is unchanged by the current flow, whereas the thinner upper nanowire at the contact point has changed significantly. It is known that the melting point of metallic nanowires decreases as their diameter decreases [28], and thus it is consistent with the thinner nanowire melting first. These observations are all consistent with Joule heating being the mechanism of welding. In Fig. 4, once the thinner nanowire has melted locally at the junction, the *I*–*V* curve is linear and, therefore, Ohmic. The weld is completed. The resistance of the two-nanowire system in this example was reduced from 2.4 × 10^12^ Ω down to 666 Ω.

Unlike Tohmyoh et al., the lack of voids along the length of the nanowires in the SEM images shown in Figs. 3 and 4 implies that a significant amount of electromigration did not occur. A relatively homogenous distribution of particles decorates the surfaces of the nanowires, particularly visible in Fig. 4, but they appear before any current flow and remain unchanged after welding so are, therefore, not from electromigration. Several others have reported the existence of surface particles on silver nanowires synthesized by the polyol method [29, 30], but their origin is still unclear at this time and requires further study.

**Fig. 5:**
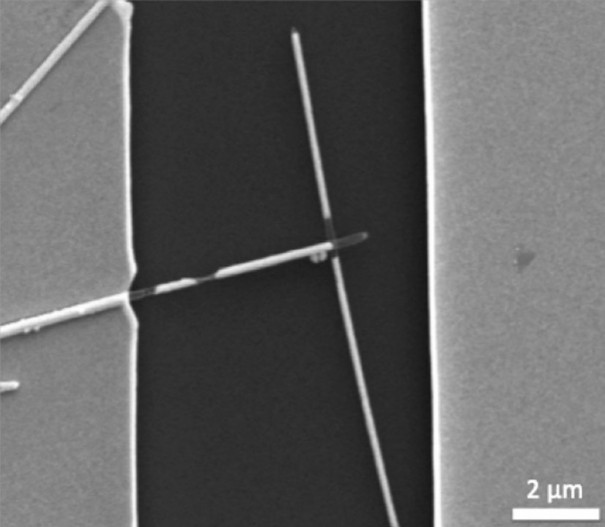
SEM image of a welded nanowire junction after applying a high enough current to cause failure. The direction of current flow is from *left* to *right*

**Fig. 6:**
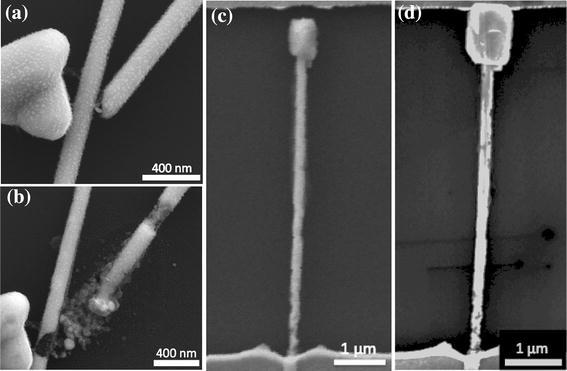
The before (**a**), and after (**b**), SEM images of the welding procedure applied to a gap junction. **c**, **d** An experiment to show that if there is a sufficient amount of silver near the junction, the gap can be closed without damaging the nanowire: a silver nanowire with a reservoir at its tip before (**c**), and after (**d**) welding

Some overlapping junctions were taken to the brink of failure to assess the failure mechanism of the wires. One such wire is shown in Fig. 5. The direction of current flow was from left to right with the current density in the thinner wire being 3.85 × 10^10^ A/m^2^. The damaged regions are concentrated around the contacts, where the resistance is larger than along the nanowires, which points to Joule heating as the mechanism for failure at these current densities. In Fig. 5, it can also be seen that there is an additional damaged area in one of the nanowires between contacts. Tohmyoh showed that when passing current through a metal nanowire, heat is not only concentrated at the contacts, but also at the midpoint along the nanowire length [14]. However, in that case, the nanowire was suspended in air, whereas here the nanowire is lying on a substrate to which heat can be transferred. We, therefore, instead expect the local temperature in the nanowire to be mainly dependent on the local resistance. The damage seen along the nanowire shown in Fig. 5 is more likely due to a notch caused by, for example, corrosion, which increases the local resistance and, therefore, Joule heating there (Fig. 6).

### Gap Junctions

The *I*–*V* responses shown in Fig. 3a, b are typical of gap junctions also, with the gap likely acting as an extension of the dielectric. In welding gap junctions, although temporary ohmic contacts were established, the nanowires did not come out of the procedure fully intact. Figure 6a, b shows a gap junction before and after the two-step welding procedure which was performed. The welding process changed the nanowire structure dramatically, with significant melting at the tip of one and the side of the other. There is also damage along the length of the nanowire on the right, which was likely caused due to excess heat created as a result of a deformity in the nanowire diameter. The junction did indeed exhibit ohmic properties; however, upon further measurements a day later, no such response was found.

To investigate whether the welding of junctions with a gap is possible without significant damage to the nanowire, we constructed an experiment. A single nanowire was found connected to an electrode on one side, and with a 400-nm gap between its other end and the electrode (Fig. 6c). The nanowire had a silver reservoir attached to the wire region closest to the gap. The first step (voltage sweeps) of the welding procedure was conducted on this nanowire, and the result is shown in Fig. 6d. Using defects in the nanowire structure, we can measure the distance between various locations on the nanowire and the bottom electrode in SEM images before and after the procedure. In the after images, it is observed that the points on the nanowire remained the same in relation to the bottom pad. Thus, physical movements such as stretching did not occur. The reservoir, however, expanded enough to close the gap. The local expansion of the reservoir points to local Joule heating as the mechanism by which gap junctions weld. It has been shown that electron discharge in gaps as large as 10 μm is possible with fairly low voltages due to a phenomenon called ion-enhanced field emission [31]. The heat caused by the electrons traversing the gap seems to melt the silver closest to the gap until the weld is complete. If, like in Fig. 6b, there is not enough silver near the tip of the nanowire to complete the weld, the generated heat causes significant melting and the destabilization of the nanowires. With the nanowire shown in Fig. 6d, however, the reservoir provided enough material for the gap to be bridged while keeping the nanowire intact. The criteria then for welding gap junctions are that the gaps must be small enough or the nanowires thick enough, so that the melted silver can traverse the gap and initiate contact. The possibility of this kind of weld is in contrast to other types of reported nanowelding, such as nanosintering, where the constituents have to be in contact for welding to take place [32].

## Conclusion

In conclusion, we have demonstrated that polyol-synthesized nanowires can welded via current flow to establish ohmic interconnects. A novel two-step electrical recipe was used to apply focused resistive heating to the contact areas to break down intermediate layers and weld overlapping nanowires without damaging the wires away from the contacts. This recipe is designed specifically for removing the intermediate layers between metal contacts, and has applicability for welding corrosion-prone nanowires or where the electrode deposition procedure used results in such layers. In particular, with devices made from metals such as silver, copper, and aluminum, oxide removal procedures can be avoided along with expensive vacuum equipment required for a contaminant-free electrode deposition processes.

Two types of junctions, overlapped and gap junctions, were identified and investigated. Overlapped junctions were reliably welded using the devised welding procedure. Gap junctions, however, were shown to be more difficult to weld requiring that an available silver reservoir traverse the gap before welding is complete.
